# Characteristics of Patients Who Visited Emergency Department: A Nationwide Population-Based Study in South Korea (2016–2018)

**DOI:** 10.3390/ijerph19148578

**Published:** 2022-07-14

**Authors:** Seok-In Hong, June-Sung Kim, Youn-Jung Kim, Dong-Woo Seo, Hyunggoo Kang, Su Jin Kim, Kap Su Han, Sung Woo Lee, Won Young Kim

**Affiliations:** 1 Department of Emergency Medicine, Asan Medical Center, University of Ulsan College of Medicine, Seoul 05505, Korea; finefigs@gmail.com (S.-I.H.); jsmeet09@gmail.com (J.-S.K.); yjkim.em@gmail.com (Y.-J.K.); leiseo@gmail.com (D.-W.S.); 2 Department of Emergency Medicine, Hanyang University College of Medicine, Seoul 04763, Korea; emer0905@gmail.com; 3 Department of Emergency Medicine, Korea University College of Medicine, Seoul 02841, Korea; icarusksj@gmail.com (S.J.K.); hanks96@hanmail.net (K.S.H.); kuedlee@korea.ac.kr (S.W.L.)

**Keywords:** direct visits, emergency department, South Korea, population-based, referrals

## Abstract

The utilization of the emergency department (ED) has been continuously increasing and has become a burden for ED resources. The aim of this study was to describe the characteristics, outcomes, common diagnoses, and disease classifications of patients who were referred to the ED. This nationwide epidemiologic study examined the data from adult patients (>18 years) who visited EDs from 1 January 2016 to 31 December 2018. Most EDs in Korea provide data from ED patients to the National Emergency Medical Center (NEMC). The disposition of ED patients was classified as discharge, admission, death, and re-transfer. From 2016 to 2018, the proportion of referred patients out of the total ED visits increased from 7.3% to 7.8%. The referred patients were older (61.1 vs. 50.5 years), had worse vital signs, longer ED lengths of stay (409.1 vs. 153.3 min), and higher admission (62.3 vs. 16.9%) and re-transfer rates (4.4 vs. 1.9%) than the direct-visit patients. Among the referred patients in the 3 years, 62.3% were hospitalized, and the most common disease classification was “disease of the digestive system” (19.8%). The most common diagnosis was pneumonia (6.0%), followed by urinary tract infection, gastrointestinal bleeding, and hepatobiliary infection. The number of patients referred to EDs is increasing, and more than 60% of referred patients are hospitalized. Detailed characteristics of these patients will be helpful for improving ED management and the distribution of medical resources.

## 1. Introduction

From the early 1990s to 2018, the increase in emergency department (ED) utilization has outpaced the growth of the general population [1,2,3,4]. Medical resource consumption and overcrowding in EDs have become public health problems [5]. Thus, efforts to construct a nationwide database on ED utilization have been made to provide trends and characteristics of patients visiting ED [3].

In Korea, data from patients who visit EDs are electronically transferred to the National Emergency Medical Center (NEMC) via a system called the National Emergency Department Information System [6]. The National Emergency Department Information System (NEDIS) is a reliable source of ED information because more than 98% of the total national EDs participate in the system. The quality of the data from the NEDIS is maintained by NEMC, a government-funded national ED control organization.

Depending on the access to specialized medical resources, three types of medical facilities exist in Korea. Primary medical facilities are small medical institutions with or without a bed that provide same-day appointments on a walk-in basis. Secondary medical facilities refer to general hospitals with beds where patients can be hospitalized for a short period of time and have multiple medical departments. Tertiary medical facilities usually refer to university hospitals for advanced medical investigation and treatment mainly for inpatients. Primary and secondary medical facilities often refer patients to the ED at higher-level hospitals. These referrals not only lead to increased costs but also potential negative consequences on the quality of care due to an unexpected increase in the usage of medical resources. The imbalance of the supply and demand in ED capacity can result in hazardous effects both on physicians and patients. Recognizing that the existing data do not provide appropriate support for resource allocation, the 72nd World Health Assembly already emphasized that member states should identify the burden of local acute illness to improve the quality of emergency care [7]. Therefore, assessing the epidemiology of patients referred to the ED is essential for preparing hospital resources, such as endoscopic bleeding control or percutaneous coronary interventions. In addition, the ED is not just an entry point to inpatient care for referred patients; new strategies may be needed to reduce the number of potentially avoidable inter-hospital transfers. In 2010, in the United States, about 1.5% of the patients in EDs were transferred to other hospitals, and the annual number of patients with transfer admissions increased [8]. If the characteristics of these patients are known, their ED utilization can be prevented or reduced by allocating an appropriate level of hospital in the initial process of seeking medical attention. However, previous studies have not focused on inter-hospital transfer outcomes or specific diagnoses [9,10], and most of the recent studies have aimed only to investigate ED visits of the self-referred patients, not the referred patients [11].

Using the NEDIS data, we conducted a nationwide epidemiologic study to investigate the characteristics, common diagnoses, disease classifications, and outcomes of adult patients who were referred to EDs. The characterization of ED visits is always an important task for healthcare improvement, and we aimed to provide detailed descriptive analyses of referred patients in Korea. Understanding the distinctive features of referrals might yield insights into addressing the public health problems and unique needs for EDs, which would help achieve the appropriate distribution of medical resources and improved management of severely ill patients in EDs. Specifically, we focused on the common diagnoses and disease classifications of referred patients, and we discuss these topics in detail.

## 2. Materials and Methods

We conducted a nationwide, cross-sectional study on the epidemiology of adult patients (>18 years) who were referred to hospital EDs from 1 January 2016 to 31 December 2018. This study was carried out in 2021 using the latest available dataset. We use the term “referral” to encompass referrals from a primary clinic to a hospital ED and transfers between hospitals. “Re-transfer” means the transfer of a patient who was initially referred from another medical facility. The terms referring to a medical facility are defined as follows: “clinic (inpatient bed <30)” and “hospital (bed <100)” refer to a primary medical facility, “general hospital (bed >99)” refers to a secondary medical facility, and “higher general hospital” refers to a tertiary medical facility.

Data were obtained from the NEDIS, a nationwide data registry in South Korea. It covers all clinical and administrative data of patients who visit EDs throughout the country. The original dataset is not open publicly and is only accessible with official permission from the NEMC. In Korea, EDs are classified into three categories according to hospital function and size: level I regional emergency centers, level II local emergency centers, and level III local emergency institutes. Since the mandatory data to be collected vary depending on the level of the ED, unlike higher-level emergency centers, most of the visits to the level III local emergency institutes lacked some data on transferring hospitals, disease categories, and severity of illness. Therefore, we mainly analyzed the visits to the level I and II emergency centers to identify the characteristics of the referred patients. The description of the total ED population, including the level III ED population, is presented only in the “Trends in ED utilization” section. We obtained official permission to use the anonymized NEDIS dataset from the NEMC. Ethical approval for this study was waived by the Asan Medical Center Institutional Review Board because of the anonymous characteristics of the data.

The dataset from the NEDIS includes information regarding age, sex, causes of visits, Korean Triage and Acuity Scale (KTAS) level, initial vital signs, insurance types, types of referring hospitals, patient dispositions, and ED length of stay (LOS) of the included ED visits. The causes of visits were categorized into two types: disease and injury. The severity of illness was based on the KTAS level and initial vital signs. In Korea, all the patients who are referred to the ED are triaged depending on their KTAS level. The scale consists of five levels and considers level 1 as the top priority, indicating the most severely ill patients. Four types of health insurance are recorded in the NEDIS data: national health insurance, government-sponsored health insurance, automobile insurance, and industry insurance. Government-sponsored health insurance (GSHI) covers almost all medical expenditures for patients with limited income and resources, similar to the Medicaid program in the United States. Patient disposition was classified into four categories: discharge, hospitalization, transfer/re-transfer to another hospital, and death. The definition of LOS was the time interval in minutes between the ED arrival and departure. Diagnoses at the ED and hospitalization were also classified by the Korean Standard Classification of Diseases, 7th revision (KCD-7) [12], which is essentially similar to the International Classification of Diseases, 10th edition (ICD-10) [13].

The data are presented as numbers with a percentage for the categorical variables and means with standard deviations (SD) for the continuous variables. All continuous variables included in this study were normally distributed. Missing data for each variable were excluded from the analysis. Since all missing data comprised less than 0.1% of each variable, they showed no statistical significance. Considering the descriptive, population-based features of this study, a routine calculation of the *p*-value was not performed. Instead, it was considered clinically significant if the difference between the direct-visit group and the referral group was more than 1%. The value was chosen deliberately based on the agreement of all authors in consideration of practical and administrative issues. All statistical analyses were performed using IBM SPSS Statistics for Windows, version 21.0 (IBM Corp., Armonk, NY, USA).

## 3. Results

### 3.1. Trends in ED Utilization

**Figure 1:**
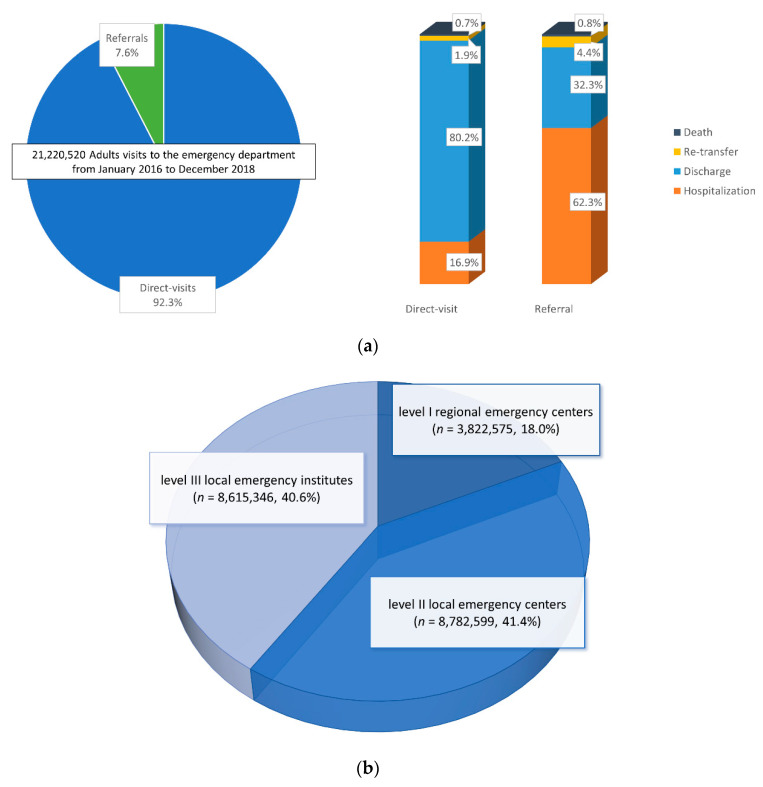
(**a**) Composition of adult emergency department (ED) visits from 2016 to 2018 by the route of visit. The ED dispositions are presented in the right 100% stacked-bar graph, comparing the direct-visit group with the referral group. (**b**) The proportion of ED visits by the level of ED is presented.

A total of 21,220,520 adult visits to the ED were recorded between January 2016 and December 2018 (3,822,575 to level I emergency centers and 8,782,599 to level II emergency centers). Among them, 1,605,304 (7.6%) were referred from other medical facilities (Figure 1).

**Figure 2:**
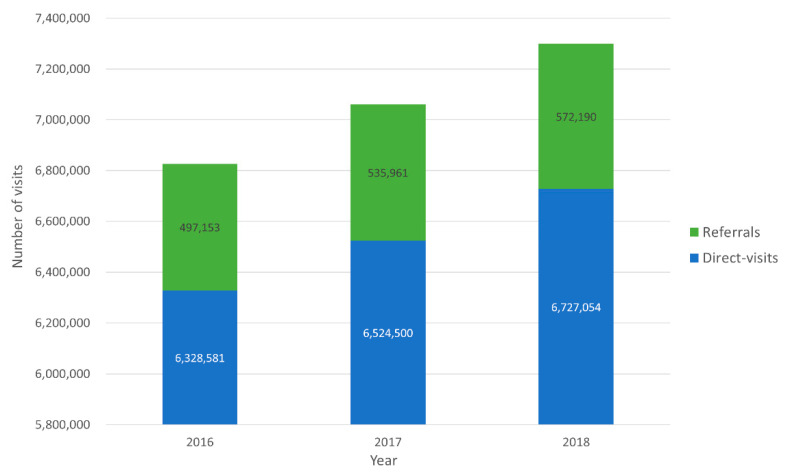
Trends in ED visits. The total number of ED visits increased from 6,844,321 to 7,305,451 and the proportion of referrals also increased from 497,153 (7.3%) to 572,190 (7.8%).

**Figure 3:**
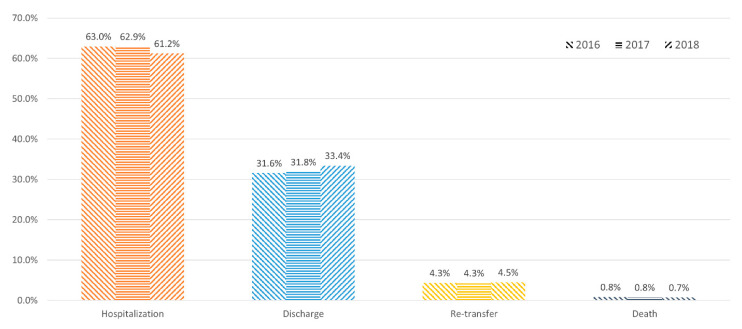
Trends in the disposition of patients who were referred. Each year is indicated with a different type of pattern. The hospitalization rate decreased from 63.0% to 61.2%, whereas the discharge rate increased from 31.6% to 33.4%. The re-transfer and death rates were almost stationary.

The trends in ED visits by referred patients from 2016 to 2018 indicate that the total number of adult ED visits increased from 6,844,321 to 7,305,451. Moreover, the proportion of referrals also steadily increased for three years: from 497,153 (7.3%) in 2016, 535,961 (7.6%) in 2017, and 572,190 (7.8%) in 2018 (Figure 2). The hospitalization rate decreased from 63.0% to 61.2%, whereas the discharge rate increased from 31.6% to 33.4% (Figure 3).

### 3.2. Differences in ED Use between Referrals and Direct Visits

Table 1 summarizes the demographic and clinical characteristics between the referred patients and direct-visit patients at level I and II emergency centers. (Supplementary Table S1). The referred patients were older than the direct-visit patients (mean age ± SD, 61.1 ± 19.0 vs. 50.5 ± 18.6 years), and the mean age difference between the groups was approximately 10 years. The proportion of the male sex was higher in the referred group (52.1% vs. 49.8%). In addition, ED visits due to disease rather than injury were more predominant in the referred group (73.9% vs. 46.4%). On the other hand, referred patients tended to have a higher severity of illness, as the proportion of a KTAS level higher than 3 was higher, and vital signs were worse in patients of the referred group than those of the direct-visit group. Moreover, the proportion of hospitalized patients was higher in the referred group (62.3% vs. 16.9%). The length of stay (LOS) in the ED was also longer in the referred group than in the direct-visit group (mean duration ± SD, 409.1 ± 753.7 vs. 153.3 ± 449.0 min). The number of GSHI beneficiaries was larger in the referred group than that in the direct-visit group (9.4% vs. 6.5%).

Some distinct differences between the direct and referred visits were observed, but none were significant enough regarding the level of ED. The proportion of patients who were referred from higher general hospitals was slightly lower in the level I emergency center group than those in the level II emergency center group (3.7% vs. 6.6%) because most of the level I emergency centers belong to higher general hospitals (Supplementary Table S1).

### 3.3. Patient Disposition in Referred Patients

Table 2 shows the demographic and clinical characteristics of referred patients according to patient disposition. Hospitalized patients accounted for the largest number (61.1%) of all referred visits. The mean age of the patients in the discharge group was 56.20 ± 19.28 (SD) years, which was younger than that in the other two groups. Disease was a predominant cause of visit in all three groups. Hospitalized and re-transferred patients tended to have a higher severity of illness, as the proportion of KTAS level 1 was higher (3.2% and 3.9%, respectively) than that of discharged patients (0.4%). Vital signs were worse, and the LOS was longer for the hospitalized (512.7 ± 772.8 min) and re-transferred patients (640.8 ± 1117.0 min) than that of the discharged patients (298.2 ± 463.2 min). In terms of insurance type, GSHI beneficiaries were more frequently found in hospitalization (11.7%) and re-transfer groups (9.1%). Most of the discharged patients were referred from clinics (43.2%), whereas others were hospitalized and re-transferred from institutions of hospital level or higher.

### 3.4. Top 10 Disease Classifications and Specific Diagnoses in Referred Patients

Table 3 describes the common disease classifications of the KCD-10. In the hospitalization group, the most common disease classification was “disease of the digestive system” (19.8%), followed by “disease of the circulatory system” (16.4%), and “injury, poisoning, and certain other consequences of external causes” (16.1%). In the re-transfer group, “injury, poisoning, and certain other consequences of external causes” (18.5%) was the most common disease classification. “Not elsewhere classified symptoms and signs” accounted for the largest proportion (21.7%) in the discharge group, immediately followed by “injury, poisoning, and certain other consequences of external causes” (21.6%). More specifically, Table 4 shows the common working diagnoses. Pneumonia was the most common diagnosis in the hospitalization (6.0%) and re-transfer (6.8%) groups, whereas gastroenteritis was the most common diagnosis in the discharge group (4.1%).

### 3.5. Differences in Survival and Non-Survival Groups in Referred Patients

The total referred patients were divided into survival and non-survival groups. The non-survival group included both death after hospitalization and death in the ED. The non-survival group accounted for 4.9% (*n* = 69,025) of the total sample population. The patients in the non-survival group were older (72.23 ± 19.28 years) than those in the survival group. Moreover, the non-survival group had a higher proportion at KTAS level 1 (20.2%), more males (58.1%), worse vital signs, and longer LOS (596.64 ± 957.75 min) than the survival group (Supplementary Table S2). The most common disease classification of the non-survival patients was “disease of the respiratory system” (22.6%), followed by “disease of the circulatory system” (21.6%) and “neoplasms” (18.8%) (Supplementary Table S3). Specifically, the most common diagnosis in the non-survival group was pneumonia (15.4%), followed by sepsis (5.6%) (Table 5).

## 4. Discussion

The results of this study indicate the following:

1. The total number of ED visits and the proportion of referrals increased from 2016 to 2018 (Figure 2).
2. The referred patients tended to have a higher severity of illness, longer ED LOS, and higher admission and re-transfer rates than direct-visit patients (Table 1).
3. The most common disease classification in the hospitalization group was “disease of the digestive system”, and the most common specific diagnosis of the referred patients resulting in admission was pneumonia, followed by urinary tract infection, gastrointestinal bleeding, and hepatobiliary infection (Table 3 and Table 4).
4. The non-survival patients accounted for 4.9% of the referred patients, with the most common disease classification and specific diagnosis being “disease of the respiratory system” and pneumonia, respectively (Table 5).

To the best of our knowledge, this is the world’s first nationwide epidemiologic study to investigate referred visits to the ED. Increasing ED visits is a global trend and outpacing population growth, reflecting a high demand for unscheduled, acute care [14,15,16]. The increase in elderly patients or younger patients with low-acuity problems might also be contributing to the increased ED visits, however, these assumptions cannot be confirmed because they were not the main focus of our study. Until recently, most studies have mainly focused on direct visits to the ED, which was regarded as an element that can be avoidable or reduced [17,18]. On the contrary, we focused on the description of the characteristics and outcomes of referred visits to the ED. We found that hospitalized patients accounted for the largest number (61.1%) of all referred visits, followed by patients who were discharged (33.6%) and re-transferred (4.2%) (Figure 1a). Although most referrals were considered appropriate or non-avoidable in a recent study investigating the appropriateness of ED visits [19,20], it was not confirmed that the discharge rate of 33.6% and re-transfer rate of 4.2% in the present study were appropriate. If the referrals are inevitable, it may also be appropriate to discuss non-avoidable ED visits and the management of severely ill patients. In a previous study, Siegfried et al. [21] emphasized that setting criteria for referral would improve the appropriateness of ED referrals from urgent care centers, and Lasserson et al. [22] suggested a staffing model to decrease the variation in referral rates among after-hour clinics. It is difficult to directly compare the results of these studies with ours due to differences in health policies among countries. However, they focused on reducing the number of unnecessary referrals in order to improve the distribution of medical resources, which we consider the next step in our study.

Emergency departments (EDs) play a unique role in the healthcare system as a place for unplanned acute care and as the chief location for admission to the hospital [23]. In the report from the 2017 National Hospital Ambulatory Medical Care Survey, the percentage of visits resulting in hospitalization was 10.4% [24]. It can be assumed that the hospitalization rate of referred patients would be higher than the overall rate, despite no mentions of referrals. In our study, more than half (61.1%) of the referred patients were hospitalized. It is obvious that the referred patients made up a large proportion of the hospitalization group due to the high severity of illness; on the other hand, the fact that they had longer ED LOS is another issue. In general, patients with severe illness required more time for diagnosis and treatment and stayed longer in the ED than those with mild illness. The high need for hospitalization may also have resulted in longer LOS because patients had to wait for inpatient beds to be allocated. Prolonged ED LOS has been associated with poor outcomes of various diseases [25], and LOS is suggested as a quality indicator in EDs recently [26]. Therefore, it is important to monitor the LOS of referred patients to maintain the quality of care in EDs.

The most common disease classification in hospitalized patients was “disease of the digestive system” (Table 3). The most common diagnosis of hospitalization was pneumonia, followed by urinary tract infection, gastrointestinal bleeding, and hepatobiliary infection (Table 4). The most common single diagnosis was pneumonia, whereas the total number of patients with disease of the digestive system (the sum of the following diagnoses, including gastrointestinal bleeding, hepatobiliary infection, acute appendicitis, and gastroenteritis) was higher than those with disease of the respiratory system. This might be because the included gastrointestinal diagnoses, except gastroenteritis, tended to have high severity requiring hospitalization, though the absolute number of patients diagnosed with pneumonia was larger than that of patients with another single diagnosis. In terms of using medical resources, gastrointestinal bleeding and hepatobiliary infection may pose a tremendous burden on healthcare utilization, because they generally require resource-consuming diagnostic or therapeutic procedures, such as an endoscopic approach for bleeding/infection control [27,28,29,30]. Considering the epidemiologic studies of ED utilization with a detailed categorization for all of the ED visits that had been limited, our results will be helpful to ED administrators in preparing manpower and room for intervention.

Additionally, we investigated the mortality rate and common causes of death of referred patients at the ED and upon admission. Our study results show that pneumonia was the leading cause of death, and the disease classification “disease of the respiratory system” comprised the largest proportion of them (Table 5). Furthermore, pneumonia was the most common cause of hospitalization and re-transfer as well (Table 4). Respiratory distress usually requires more concentrated hospital medical resources, equipment, and space for care [31]. Moreover, the pneumonia attack rate increased rapidly with age. The rate was 12 per 1000 in adults aged 75 and older and 33 per 1000 for nursing home residents [32]. Pneumonia was also the most common diagnosis among survived patients, followed by urinary tract infection and hepatobiliary infection. Regardless of survival, infectious disease was in the top three diseases. Given the recent COVID-19 pandemic, the number of patients with respiratory disease may have increased even more, and thus, mobilizing resources to save lives has become more important.

Another distinctive result of this study was that the proportion of GSHI beneficiaries was higher in the referred group than that in the other groups (Table 1). This brings multiple hypotheses into consideration: the possibilities of the high burden of illness in low-income patients, the incapability of GSHI-designated hospitals to cover the high severity of illnesses, or simply the reflection of frequent ED use by GSHI beneficiaries. Associations between ED use and health insurance coverage were already reported; expanded insurance coverage induced more ED visits even if the relationship was not straightforward [33]. Although it does not make up much of the proportion of ED visits, the visits by GSHI beneficiaries should be considered in the aspect of the role of the ED as a social safety net.

The design of the nationwide study, the large population, and the data collection over three years are the strengths of our study. However, this study has several limitations. First, we were unable to make a proposal for improving the referral system or utilizing medical resources. This study presents detailed descriptive results of an epidemiologic study; however, we could not draw conclusions to help clinical decisions. In addition, only a few studies have investigated ED referrals, and it was difficult to use other similar studies as the basis for this present study due to differences in the study population. Although we achieved the goal of understanding the nature of referrals in EDs, future research is essential for suggesting an intervention, such as a decision model in health policies. Second, we mainly analyzed the data from the regional or local medical emergency centers because essential data from local emergency institutes were insufficient. Therefore, the generalization of these results to small-sized EDs may not be appropriate. Moreover, our study findings represent ED utilization in only South Korea. One should be careful in interpreting our findings in other countries in an extended way. Third, the NEDIS data do not provide unique patient identifiers; some visits represented repeated visits for the same patients. Fourth, we only investigated the primary diagnoses of the included visits. A patient could be diagnosed with more than one disease and sometimes be diagnosed with a disease that they already had, such as malignancies. However, we regard the primary diagnosis as the main diagnosis or a problem that the patient had, which reflects the cause of the ED visit. Lastly, we cannot identify the exact hospitals or primary care clinics from which the patients were referred. Increases in ED visits and referrals may reflect a deteriorating primary care infrastructure and fragmentation of the healthcare delivery system. However, these considerations are beyond the scope and objective of our study, and further investigation will be required. Despite these limitations, the national trends found in this study provide important insights into the characteristics and outcomes of referred patients in EDs.

## 5. Conclusions

In South Korea, ED referrals increased from 7.3% to 7.8% in 3 years. Referred patients tended to have a high severity of illness, with a 61.1% hospitalization rate, which also resulted in longer LOS that could compromise the quality of care in EDs. In hospitalized patients, the most common disease classification was “disease of the digestive system”. In terms of specific diagnoses, pneumonia and infectious diseases were the leading cause of hospitalization, re-transfer, and death. Knowing common diseases can reduce the burden on healthcare utilization by appropriately mobilizing medical resources. Future nationwide research is essential for proposing an intervention to improve the distribution of medical resources by assessing emergency care needs and the ability of the healthcare setting.

## Figures and Tables

**Table 1:** Clinical characteristics and outcomes of direct visits vs. referrals at level I and II emergency centers.

	Level I + II Emergency Centers		
Variables	Total (*n* = 21,220,520)	Direct Visit (*n* = 19,580,135, 92.3%)	Referral (*n* = 1,605,304, 7.6%)
Age (year, ±SD)	51.3 (±18.8)	50.5 (±18.6)	61.1 (±19.0)
Sex, male (%)	10,596,788 (49.9)	9,741,546 (49.8)	836,913 (52.1)
Cause of visit, *n* (%)			
Disease	10,277,717 (48.4)	9,086,281 (46.4)	1,185,825 (73.9)
Injury	3,854,361 (18.2)	3,590,875 (18.3)	261,301 (16.3)
KTAS, *n* (%)			
Level 1	185,829 (0.9)	147,330 (0.8)	37,758 (2.4)
Level 2	895,053 (4.2)	673,795 (3.4)	220,166 (13.7)
Level 3	4,872,467 (23.0)	4,111,396 (21.0)	756,490 (47.1)
Level 4	6,063,932 (28.6)	5,699,782 (29.1)	358,121 (22.3)
Level 5	1,653,984 (7.8)	1,590,365 (8.1)	60,443 (3.8)
Vital sign (±SD)			
SBP	133.2 (±24.3)	133.4 (±24.0)	131.9 (±26.9)
DBP	79.8 (±14.6)	80.1 (±14.4)	78.0 (±16.0)
PR	85.1 (±17.1)	84.8 (±16.8)	87.4 (±19.1)
RR	19.4 (±2.8)	19.4 (±2.7)	19.7 (±3.2)
BT	36.8 (±0.7)	36.8 (±0.7)	36.8 (±0.7)
LOS (min ± SD)	172.6 (±484.1)	153.3 (±449.0)	409.1 (±753.7)
Insurance type, *n* (%)			
National	18,074,424 (85.2)	16,690,937 (85.2)	1,363,408 (84.9)
GSHI	1,421,898 (6.7)	1,269,006 (6.5)	150,707 (9.4)
Automobile	1,014,934 (4.8)	963,915 (4.9)	50,022 (3.1)
Industry	91,658 (0.4)	82,229 (0.4)	9232 (0.6)
None	467,046 (2.2)	438,750 (2.2)	22,684 (1.4)
Referred from			
Higher general hospital			74,691 (4.7)
General hospital			448,010 (27.9)
Hospital			467,888 (29.1)
Clinic			382,857 (23.9)
Patient disposition			7649 (0.5)
Discharge	16,239,842 (76.5)	15,711,986 (80.2)	518,729 (32.3)
Hospitalization	4,312,635 (20.3)	3,309,124 (16.9)	1,000,451 (62.3)
Transfer	434,297 (2.0)	363,524 (1.9)	70,315 (4.4)
Death	143,218 (0.7)	129,875 (0.7)	12,390 (0.8)

Data are presented as numbers with percentages for categorical variables and means with standard deviations for continuous variables. Abbreviations: BT, body temperature; DBP, diastolic blood pressure; ED, emergency department; GSHI, government-sponsored health insurance; KTAS, Korean Triage and Acuity Scale; LOS, length of stay; PR, pulse rate; RR, respiratory rate; SBP, systolic blood pressure; SD, standard deviations.

**Table 2:** Clinical characteristics based on the patient dispositions in referred visits.

Variables	Discharge (*n* = 470,589, 33.6%)	Hospitalization (*n* = 855,022, 61.1%)	Re-Transfer (*n* = 59,274, 4.2%)
Age (year, ±SD)	56.2 (±19.3)	63.1 (±18.3)	65.9 (±17.8)
Sex, male (%)	231,634 (49.2)	456,291 (53.4)	31,777 (53.6)
Cause of visit, *n* (%)			
Disease	370,366 (78.7)	718,248 (84.0)	48,461 (81.8)
Injury	99,381 (21.1)	134,712 (15.8)	10,613 (17.9)
KTAS, *n* (%)			
Level 1	2073 (0.4)	27,285 (3.2)	2293 (3.9)
Level 2	42,172 (9.0)	159,988 (18.7)	10,838 (18.3)
Level 3	220,449 (46.8)	487,948 (57.1)	31,296 (52.8)
Level 4	175,080 (37.2)	155,589 (18.2)	12,654 (21.3)
Level 5	29,989 (6.4)	22,177 (2.6)	1983 (3.3)
Vital sign (±SD)			
SBP	135.5 (±24.7)	130.4 (±27.5)	129.2 (±28.5)
DBP	80.2 (±14.8)	77.1 (±16.4)	76.5 (±16.9)
PR	85.2 (±17.7)	88.4 (±19.6)	89.1 (±20.2)
RR	19.2 (±2.6)	19.9 (±3.5)	20.0 (±3.5)
BT	36.8 (±0.6)	36.9 (±0.7)	36.8 (±0.8)
LOS (min ±SD)	298.2 (±463.2)	512.7 (±772.8)	640.8 (±1117.0)
Insurance type, *n* (%)			
National	411,141 (87.4)	734,084 (85.9)	49,008 (82.7)
GSHI	38,467 (8.2)	78,253 (9.1)	6949 (11.7)
Automobile	10,404 (2.2)	25,089 (2.9)	2040 (3.4)
Industry	940 (0.2)	5147 (0.6)	174 (0.3)
None	6761 (1.4)	9629 (1.1)	633 (1.1)
Referred from			
Higher general hospital	11,964 (2.5)	59,548 (7.0)	2413 (4.1)
General hospital	102,240 (21.7)	322,565 (37.7)	17,751 (29.9)
Hospital	140,770 (29.9)	289,802 (33.9)	29,142 (49.2)
Clinic	203,353 (43.2)	169,055 (19.7)	8706 (14.7)

Data are presented as numbers with percentages for categorical variables and means with standard deviations for continuous variables. Abbreviations: BT, body temperature; DBP, diastolic blood pressure; GSHI, government-sponsored health insurance; KTAS, Korean Triage and Acuity Scale; LOS, length of stay; PR, pulse rate; RR, respiratory rate; SBP, systolic blood pressure; SD, standard deviations.

**Table 3:** Top 10 disease classifications based on patient dispositions in referred visits.

Discharge (*n* = 470,589, 33.6%)		Hospitalization (*n* = 855,022, 61.1%)		Re-Transfer (*n* = 59,274, 4.2%)	
Symptoms, signs, and abnormal clinical and laboratory findings, NEC	101,917 (21.7)	Diseases of the digestive system	168,783 (19.8)	Injury, poisoning, and certain other consequences of external causes	10,983 (18.5)
Injury, poisoning, and certain other consequences of external causes	101,347 (21.6)	Diseases of the circulatory system	140,097 (16.4)	Diseases of the digestive system	9324 (15.7)
Diseases of the digestive system	39,405 (8.4)	Injury, poisoning, and certain other consequences of external causes	136,861 (16.1)	Symptoms, signs, and abnormal clinical and laboratory findings, NEC	7375 (12.5)
Diseases of the circulatory system	34,306 (7.3)	Diseases of the respiratory system	108,876 (12.8)	Diseases of the circulatory system	7366 (12.4)
Diseases of the genitourinary system	33,128 (7.1)	Symptoms, signs, and abnormal clinical and laboratory findings, NEC	69,661 (8.2)	Diseases of the respiratory system	6716 (11.3)
Diseases of the respiratory system	32,211 (6.9)	Diseases of the genitourinary system	57,182 (6.7)	Neoplasms	5551 (9.4)
Certain infectious and parasitic diseases	26,927 (5.7)	Neoplasms	48,104 (5.6)	Diseases of the genitourinary system	3530 (6.0)
Neoplasms	18,236 (3.9)	Certain infectious and parasitic diseases	34,319 (4.0)	Certain infectious and parasitic diseases	1788 (3.0)
Diseases of the musculoskeletal system and connective tissue	14,163 (3.0)	Diseases of the musculoskeletal system and connective tissue	15,252 (1.8)	Diseases of the musculoskeletal system and connective tissue	1239 (2.1)
Diseases of the nervous system	13,037 (2.8)	Pregnancy, childbirth, and the puerperium	14,419 (1.7)	Endocrine, nutritional, and metabolic diseases	1157 (2.0)

Data are presented as numbers with percentages. Diseases were classified according to the Korean Standard Classification of Diseases, 10th revision (KCD-10). Abbreviations: NEC, not elsewhere classified.

**Table 4:** Top 10 specific diagnoses based on patient dispositions in referred visits.

Discharge (*n* = 470,589, 33.6%)		Hospitalization (*n* = 855,022, 61.1%)		Re-Transfer (*n* = 59,271, 4.2%)	
Gastroenteritis	19,289 (4.1)	Pneumonia	51,685 (6.0)	Pneumonia	3993 (6.8)
Dizziness	13,462 (2.9)	Urinary tract infection	23,666 (2.8)	Acute appendicitis	2080 (3.5)
Chest pain	10,980 (2.3)	Gastrointestinal bleeding	22,974 (2.7)	Urinary tract infection	1574 (2.7)
Pneumonia	9526 (2.0)	Hepatobiliary infection	21,745 (2.6)	Fever	1155 (1.9)
Head trauma	9083 (1.9)	Ischemic stroke	21,493 (2.5)	Dyspnea	997 (1.7)
Abdominal pain	8981 (1.9)	Acute appendicitis	20,040 (2.3)	Traumatic subdural hemorrhage	909 (1.5)
Fever	8471 (1.8)	Acute myocardial infarction	17,063 (2.0)	Femur fracture	825 (1.4)
Ureteric stone	8466 (1.8)	Traumatic subdural hemorrhage	10,933 (1.3)	Ischemic stroke	734 (1.2)
Headache	7062 (1.5)	Fever	10,852 (1.3)	Gastrointestinal bleeding	599 (1.0)
Urinary tract infection	6895 (1.4)	Gastroenteritis	8821 (1.0)	Malignant neoplasm of bronchus or lung	530 (0.9)

Data are presented as numbers and percentages.

**Table 5:** Top 10 common diagnoses in referred visits of survival and non-survival patients.

Survival Patients (*n* = 1,327,156, 94.8%)		Non-Survival Patients (69,025, 4.9%)	
Pneumonia	58,296 (4.4)	Pneumonia	10,596 (15.4)
Urinary tract infection	31,517 (2.4)	Sepsis	3899 (5.6)
Hepatobiliary infection	29,633 (2.2)	Cardiac arrest	3761 (5.4)
Acute appendicitis	28,545 (2.2)	Malignant neoplasm of liver cell carcinoma	1537 (2.2)
Gastroenteritis	26,135 (2.0)	Acute myocardial infarction	1349 (2.0)
Ischemic stroke	21,233 (1.6)	Malignant neoplasm of the bronchus or lung	1229 (1.8)
Acute myocardial infarction	15,354 (1.2)	Traumatic subdural hemorrhage	1135 (1.6)
Femur fracture	14,504 (1.1)	Respiratory failure/pulmonary edema	954 (1.4)
Dizziness	13,462 (1.0)	Heart failure	829 (1.2)
Chest pain	11,385 (0.9)	Acute kidney injury	819 (1.2)

Data are presented as numbers and percentages.

## Data Availability

Data are available from the corresponding author upon reasonable request and with the permission of the National Emergency Medical Center of Korea.

## Supplementary Materials

The following supporting information can be downloaded at: https://www.mdpi.com/article/10.3390/ijerph19148578/s1, Table S1: Clinical characteristics and outcomes of direct visits vs. referrals stratified by the levels of emergency centers; Table S2: Comparison of characteristics in referred visits by survival; Table S3: Disease classifications in referred visits by survival.
