# Supplementary material for: Characteristics of Patients Who Visited Emergency Department: A Nationwide Population-Based Study in South Korea (2016–2018)
Source: Int J Environ Res Public Health. 2022 Jul 14;19(14):8578. doi: 10.3390/ijerph19148578 (PMC9316116; doi:10.3390/ijerph19148578)
Supplement: Supplementary file 1 [file ijerph-19-08578-s001.zip › ijerph-1732558-supplementary.pdf]

Supplementary Table S1. Clinical characteristics and outcomes of direct-visits vs. referrals stratified by the levels of emergency centers.

Variables	Level I regional emergency center (n = 3,822,575)		Level II local emergency center (n = 8,782,599)	
	Direct-visit (n = 3,190,483, 83.5%)	Referral (n = 628,468, 16.4%)	Direct-visit (n = 8,001,246, 91.1%)	Referral (n = 770,871, 8.8%)
Age (year, ±SD)	51.6 (±18.7)	60.9 (±18.7)	50.5 (±18.8)	61.0 (±19.0)
Sex, male (%)	1,596,087 (50.0)	332,836 (53.0)	3,916,315 (48.9)	394,885 (51.2)
Cause of visit, n (%)				
Disease	2,332,663 (73.1)	514,348 (81.8)	561,587 (70.2)	635,351 (82.4)
Injury	856,757 (26.9)	113,923 (18.1)	2,286,043 (28.6)	132,522 (17.2)
KTAS, n (%)				
Level 1	49,716 (1.6)	23,283 (3.7)	90,545 (1.1)	14,013 (1.8)
Level 2	259,450 (8.1)	116,474 (18.5)	389,726 (4.9)	100,244 (13.0)
Level 3	1,273,254 (39.9)	336,132 (53.5)	2,574,234 (32.2)	406,771 (52.8)
Level 4	1,302,739 (40.8)	134,434 (21.4)	3,832,761 (47.9)	209,898 (27.2)
Level 5	304,113 (9.5)	18,000 (2.9)	1,019,618 (12.7)	36,959 (4.8)
Vital sign (±SD)				
SBP	135.6 (±25.0)	132.8 (±27.5)	132.5 (±23.5)	131.2 (±26.3)
DBP	80.7 (±15.2)	78.4 (±16.6)	79.8 (±14.1)	77.7 (±15.6)
PR	86.0 (±17.7)	87.7 (±19.5)	84.4 (±16.3)	87.1 (±18.9)
RR	19.2 (±3.1)	19.5 (±3.4)	19.5 (±2.6)	19.7 (±3.1)
BT	36.7 (±0.7)	36.8 (±0.7)	36.8 (±0.7)	36.9 (±0.7)
LOS (min ±SD)	246.1 (±519.6)	465.1 (±741.2)	192.2 (±437.5)	431.7 (±692.6)
Insurance type, n (%)				
National	2,792,246 (87.5)	543,341 (86.5)	6,841,329 (85.5)	662,432 (85.9)
GSHI	189,279 (5.9)	53,238 (8.5)	513,921 (6.4)	72,456 (9.4)
Automobile	135,964 (4.3)	18,119 (2.9)	404,900 (5.1)	19,888 (2.6)
Industry	7,027 (0.2)	2,554 (0.4)	22,341 (0.3)	3,752 (0.5)
None	56,223 (1.8)	9,236 (1.5)	162,009 (2.0)	8,102 (1.1)
Referred from				
Higher general hospital		23,316 (3.7)		51,135 (6.6)
General hospital		239,323 (38.1)		207,922 (27.0)
Hospital		203,070 (32.3)		264,213 (34.3)
Clinic		152,754 (24.4)		229,643 (29.8)
Patient disposition				
Discharge	2,400,640 (75.2)	208,563 (33.2)	6,297,858 (78.7)	262,026 (34.0)
Hospitalization	706,667 (22.1)	387,295 (61.6)	1,485,815 (18.6)	467,727 (60.7)

Transfer	50,914 (1.6)	26,466 (4.2)	133,826 (1.7)	32,805 (4.3)
Death	22,994 (0.7)	5,449 (0.9)	58,804 (0.7)	5,850 (0.8)

Data are presented as numbers with percentages for categorical variables and means with standard deviations for continuous variables.

Abbreviations: BT, body temperature; DBP, diastolic blood pressure; ED, emergency department; GSHI, government-sponsored health insurance; KTAS, Korean Triage and Acuity Scale; LOS, length of stay; PR, pulse rate; RR, respiratory rate; SBP, systolic blood pressure; SD, standard deviations

Supplementary Table S2. Comparison of characteristics in referred visits by survival.

Variables	Survival patients (n = 1,327,156, 94.8%)	Non-survival patients (n = 69,025, 4.9%)
Age (year, ±SD)	60.4 (±18.9)	72.2 (±13.7)
Sex, male (%)	685,978 (51.7)	70,079 (58.1)
Cause of visit, n (%)		
Disease	1,083,576 (81.6)	63,595 (92.1)
Injury	240,633 (18.1)	5,202 (7.5)
KTAS, n (%)		
Level 1	23,326 (1.8)	13,921 (20.2)
Level 2	194,544 (14.7)	21,843 (31.6)
Level 3	714,331 (53.8)	27,398 (39.7)
Level 4	339,143 (25.6)	4,373 (6.3)
Level 5	52,902 (4.0)	1,286 (1.9)
Vital sign (±SD)		
SBP	132.64 (±26.33)	117.09 (±32.79)
DBP	78.40 (±15.74)	70.08 (±19.70)
PR	86.90 (±18.71)	97.30 (±24.37)
RR	19.56 (±3.09)	21.59 (±5.27)
BT	36.83 (±0.70)	36.67 (±0.93)
LOS (min ±SD)	439.04 (±699.47)	596.64 (±957.75)
Insurance type, n (%)		
National	1,145,475 (86.3)	57,852 (83.8)
GSHI	116,576 (8.8)	8,579 (12.4)
Automobile	36,657 (2.8)	1,257 (1.8)
Industry	6,111 (0.5)	177 (0.3)
None	16,399 (1.2)	889 (1.3)
Referred from		
Higher general hospital	67,599 (5.1)	6,747 (9.8)
General hospital	420,333 (31.7)	26,291 (38.1)
Hospital	434,071 (32.7)	31,661 (45.9)
Clinic	378,437 (28.5)	3,138 (4.6)

Data are presented as numbers with percentages for categorical variables and means with standard deviations for continuous variables.

Abbreviations: BT, body temperature; DBP, diastolic blood pressure; ED, emergency department; GSHI, government-sponsored health insurance; KTAS, Korean Triage and Acuity Scale; LOS, length of stay; PR, pulse rate; RR, respiratory rate; SBP, systolic blood pressure; SD, standard deviations

Supplementary Table S3. Disease classifications in referred visits by survival.

Survival patients (n = 1,327,156, 94.8%)		Non-survival patients (69,025, 4.9%)	
Injury, poisoning, and certain other consequences of external causes	244,260 (18.7)	Diseases of the respiratory system	15,464 (22.6)
Diseases of the digestive system	203,597 (15.6)	Diseases of the circulatory system	14,826 (21.6)
Diseases of the circulatory system	176,696 (13.5)	Neoplasms	12,912 (18.8)
Symptoms, signs, and abnormal clinical and laboratory findings, NEC	130,900 (10.0)	Diseases of the digestive system	5,726 (8.4)
Diseases of the respiratory system	130,265 (10.0)	Certain infectious and parasitic diseases	4,655 (6.8)
Diseases of the genitourinary system	92,461 (7.1)	Injury, poisoning, and certain other consequences of external causes	4,302 (6.3)
Neoplasms	79,661 (6.1)	Symptoms, signs, and abnormal clinical and laboratory findings, NEC	3,971 (5.8)
Certain infectious and parasitic diseases	65,978 (5.1)	Diseases of the genitourinary system	3,083 (4.5)
Diseases of the musculoskeletal system and connective tissue	31,576 (2.4)	Endocrine, nutritional, and metabolic diseases	974 (1.4)
Diseases of the nervous system	31,213 (2.4)	Diseases of the nervous system	776 (1.1)

Data are presented as numbers with percentages. Diseases were classified by the Korean Standard Classification of Diseases, 10th revision (KCD-10).

Abbreviations: NEC, not elsewhere classified
